# Supplementary material for: Inference of Molecular Regulatory Systems Using Statistical Path-Consistency Algorithm
Source: Entropy (Basel). 2022 May 13;24(5):693. doi: 10.3390/e24050693 (PMC9142129; doi:10.3390/e24050693)
Supplement: Supplementary file 1 [file entropy-24-00693-s001.zip › Supple.pdf]

Supplementary Information

Inference of molecular regulatory systems using statistical path-consistency algorithm

1. Information theory

Compared with correlation coefficient, the mutual information (MI) from information theory is able to describe the nonlinear dependence between two random variables. Let X be a random variable with density function $p(x)$. We first introduce the concept of entropy, which in statistical mechanics is the measure of a system's thermal energy per unit temperature that is unavailable for doing useful work. The entropy $H(X)$ of X is defined by

$$\begin{aligned} H(X) &= -\sum_{i=1}^{N_x} p(x_i)\log p(x_i) \\ H(X) &= -\int_{\Omega_X} p(x)\log p(x)dx, \end{aligned} \tag{1.1}$$

for discrete and continuous random variables, respectively, where $x_1, ..., x_{N_x}$ are samples of random variable X in the discrete case, and Ω_X is the integral range of random variable X.. In addition, for two random variables X and Y, the joint entropy $H(X, Y)$ is defined by

$$\begin{aligned} H(X,Y) &= -\sum_{i=1}^{N_x}\sum_{j=1}^{N_y} p(x_i, y_j)\log p(x_i, y_j) \\ H(X,Y) &= -\int\int_{\Omega_X\times\Omega_Y} p(x,y)\log p(x,y)dxdy, \end{aligned} \tag{1.2}$$

for discrete and continuous random variables, respectively, where $p(x, y)$ is the joint density function of random variables X and Y, $y_1, ..., y_{N_y}$ are samples of random variable Y, and Ω_X and Ω_Y are the integral range of X and Y, respectively.

Mutual information measures the nonlinear dependency between two random variables. For discrete variables X and Y, it can be calculated from

$$MI(X,Y) = \sum_{i=1}^{N_x}\sum_{j=1}^{N_y} p(x_i, y_j)\log\frac{p(x_i, y_j)}{p(x_i)p(y_j)}, \tag{1.3}$$

where $p(x)$ and $p(y)$ are marginal density functions of variables X and Y, respectively. In addition, mutual information can be measured in terms of entropies as

$$MI(X,Y) = H(X) + H(Y) - H(X,Y). \tag{1.4}$$

If the value of mutual information is zero, these two random variables are independent of each other. However, a larger value of mutual information generally suggests a closer relationship between the two random variables.

For a system with more random variables, the strong dependence relationship of two random variables may be caused by the third random variable. To address this issue, conditional mutual information (CMI) measures conditional dependency between two random variables under the condition of the third variable. The value of CMI between variables X and Y given Z is defined by

$$\text{CMI}(X,Y|Z) = H(X,Z) + H(Y,Z) - H(Z) - H(X,Y,Z), \tag{1.5}$$

where $H(X, Y, Z)$ is the joint entropy of these three random variables, defined by

$$
\begin{aligned}
H(X,Y,Z) &= -\sum_{i=1}^{N_x}\sum_{j=1}^{N_y}\sum_{k=1}^{N_z} p(x_i, y_j, z_k)\log p(x_i, y_j, z_k) \\
H(X,Y,Z) &= -\int\int\int_{\Omega_X\times\Omega_Y\times\Omega_Z} p(x,y,z)\log p(x,y,z)dxdydz, \qquad (1.6)
\end{aligned}
$$

and $p(x, y, z)$ is the joint density function of random variables X, Y and Z, and $z_1, ..., z_{N_z}$ are samples of random variable Z. In addition, Ω_X, Ω_Y and Ω_Z are the integral range of X, Y and Z, respectively. If variables X and Y are independent of each other under the condition of variable Z, then $\text{CMI}(X, Y|Z) = 0$.

For discrete random variables, the CMI can also be calculated from

$$
\text{CMI}(X,Y|Z) = -\sum_{i=1}^{N_x}\sum_{j=1}^{N_y}\sum_{k=1}^{N_z} p(x_i, y_j, z_k)\log\frac{p(x_i, y_j|z_k)}{p(x_i|z_k)p(y_j|z_k)}, \qquad (1.7)
$$

where $p(x|z)$ and $p(y|z)$ are the density functions of random variables X and Y under the given third random variable Z, respectively, and $p(x, y|z)$ is the joint probability of the random variables X and Y under the condition of given Z. If variables X and Y are independent of each other under the condition of variable Z, then $\text{CMI}(X, Y|Z) = 0$.

For a regulatory network of m genes, the activity of gene X_i is measured by the expression levels at different time points $(x_{i1}, ..., x_{in})$. We can calculate the frequency of the expression data and then use the frequency to approximate the mutual information [**?**]. To this end, we first uniformly divide the interval $[\min_j(x_{ij}), \max_j(x_{ij})]$ into k subintervals, and compute the frequency of the expression data falling into the subinterval q, and then approximate the probability by using

$$
f_{iq} \approx \frac{f_{iq}}{q}, \quad i = 1, ...n, q = 1, ..., k.
$$

Similar formulas can be derived for calculating the joint probability $p(x, y)$ of two random variables. Then we can use these approximated probabilities to calculate MI.

We can also calculate mutual information by using the assumed probability density functions. A particular case is the Gaussian kernel probability density function [**? ?**]. The kernel density estimation is a non-parametric method to estimate the probability density function of a random variable. It is a fundamental data smoothing problem where inferences about the population are made. The probability density estimator is given by

$$
P(X_i) = \frac{1}{N}\sum_{j=1}^{N}\frac{1}{(2\pi)^{n/2}|C|^{n/2}}\exp\left(-\frac{1}{2}(X_j - X_i)^T C^{-1}(X_j - X_i)\right),
$$

where C is the covariance matrix of variable X, $|C|$ is the determinant of matrix C, N is the number of samples, and n is the number of variables in X. Then the entropy of variable X can be calculated by

$$
H(X) = \frac{1}{2}n\log(2\pi e)|C|,
$$

and the mutual information and conditional mutual information are given by

$$
\begin{aligned}
\text{MI}(X,Y) &= \frac{1}{2}\log\frac{|C(X)||C(Y)|}{|C(X,Y)|}, \qquad (1.8) \\
\text{CMI}(X,Y|Z) &= \frac{1}{2}\log\frac{|C(X,Z)||C(Y,Z)|}{|C(Z)||C(X,Y,Z)|}, \qquad (1.9)
\end{aligned}
$$

where $|C(X)|$, $|C(Y)|$, and $|C(Z)|$ are the variance of random variables X, Y, and Z, respectively, for the case of one random variable. In addition, $|C(X, Y)|$ is the covariance of random variables X and Y, and $|C(X, Y, Z)|$ is the determinant of the covariance matrix of random variables X, Y and Z.

When using the mutual information to measure the correlation between two variables, the correlation between two random variables is often overestimated, resulting in networks with more false positive edges. However, when using conditional mutual information to measure the correlation between two variables, the correlation between these two variables is often underestimated, resulting in networks with more false negative edges. To address this issue, part mutual information (PMI) is proposed to reduce both the false positive rate and false negative rate [**?**][**?**].

The partial independence of the random variables X and Y under the given variable Z is defined by [**?**]:

$$p^*(x|z)p^*(y|z) = p(x,y|z) \tag{1.10}$$

where

$$p^*(x|z) = \sum_y p(x|z,y)p(y),$$

$$p^*(y|z) = \sum_y p(y|z,x)p(x),$$

where $p(x|z,y)$ is the conditional density of X given (Y,Z).

According to the definition of partial independence formula (**??**), part mutual information is defined as:

$$\mathrm{PMI}(X,Y|Z) = \sum_{x,y,z} p(x,y,z) log \frac{p(x,y|z)}{p^*(x|z)p^*(y|z)}. \tag{1.11}$$

References

[1] Guo, X., Zhang, H., Tian, T.: Development of stock correlation networks using mutual information and financial big data. PLoS ONE, 2018, 13, e0195941.

[2] Basso, K.; Margolin, A.A.; Stolovitzky, G.; Klein, U.; Dallafavera, R.; Califano, A. Reverse engineering of regulatory networks in human b cells. Nat Genet, 2005, **37**, 382–390.

[3] Zhang, X., Zhao, X., He, K., Lu, L., Cao, Y., Liu,J., Hao, J., Liu, Z., Chen, L.: Inferring gene regulatory networks from gene expression data by path consistency algorithm based on conditional mutual information. Bioinformatics, 2012, **28**, 98–104.

[4] Janzing, D., Balduzzi, D., Grosse-Wentrup, M.,Sch?lkopf, B.: Quantifying causal influences. Annals of Statistics, 2013, **41**, 2324–2358.

[5] Zhao, J., Zhou, Y., Zhang, X., Chen, L.: Part mutual information for quantifying direct associations in networks. Proc Natl Acad Sci U S A, 2016, **113**, 5130-5135.
